# ERLNs augment simultaneous delivery of GFSV into PC-3 cells: Influence of drug combination on SDH, GPX-4, 5α-RD, and cytotoxicity

**DOI:** 10.32604/or.2024.054537

**Published:** 2025-03-19

**Authors:** RIYAD F. ALZHRANI, LAMA BINOBAID, ABDULAZIZ A. ALORAINI, MESHAL S. ALSAHLI, AHMED H. BAKHEIT, HANADI H. ASIRI, SABRY M. ATTIA, ALI ALHOSHANI, GAMALELDIN I. HARISA

**Affiliations:** 1 Department of Pharmaceutics, College of Pharmacy, King Saud University, Riyadh, 11451, Saudi Arabia; 2 Department of Pharmacology and Toxicology, College of Pharmacy, King Saud University, Riyadh, 11451, Saudi Arabia; 3 Department of Pharmaceutical Chemistry, College of Pharmacy, King Saud University, Riyadh, 11451, Saudi Arabia

**Keywords:** Prostate cancer (PCA), Nanoparticle biocompatibility, Molecular docking simulation (MDS), Apoptosis, Drug repurposing

## Abstract

**Objective:**

Prostate cancer (PCA) is the second most widespread cancer among men globally, with a rising mortality rate. Enzyme-responsive lipid nanoparticles (ERLNs) are promising vectors for the selective delivery of anticancer agents to tumor cells. The goal of this study is to fabricate ERLNs for dual delivery of gefitinib (GF) and simvastatin (SV) to PCA cells.

**Methods:**

ERLNs loaded with GF and SV (ERLNGFSV) were assembled using bottom-up and top-down techniques. Subsequently, these ERLN cargoes were coated with triacylglycerol, and phospholipids and capped with chitosan (CS). The ERLNGFSV, and CS engineered ERLNGFSV (CERLNGFSV) formulations were characterized for particle size (PS), zeta potential (ZP), and polydispersity index (PDI). The biocompatibility, and cytotoxicity of the plain and GF plus SV-loaded ERLN cargoes were assessed using erythrocytes and PC-3 cell line. Additionally, molecular docking simulations (MDS) were conducted to examine the influence of GF and SV on succinate dehydrogenase (SDH), glutathione peroxidase-4 (GPX-4), and 5α-reductase (5α-RD).

**Results:**

These results showed that plain, ERLNGFSV, and CERLNGFSV cargoes have a nanoscale size and homogeneous appearance. Moreover, ERLNGFSV and CERLNGFSV were biocompatible, with no detrimental effects on erythrocytes. Treatment with GF, SV, GF plus SV, ERLNGFSV, and CERLNGFSV significantly reduced the viability of PC-3 cells compared to control cells. Particularly, the blend of GF and SV, as well as ERLNGFSV and CERLNGFSV augmented PC-3 cell death. Also, treating PC-3 cells with free drugs, their combination, ERLNGFSV, and CERLNGFSV formulations elevated the percentage of apoptotic cells. MDS studies demonstrated that GF and SV interact with the active sites of SDH, GPX-4, and 5α-reductase.

**Conclusions:**

This study concludes that SVGF combination and ERLNs loading induce particular delivery, and synergism on PC-3 death through action on multiple pathways involved in cell proliferation, and apoptosis, besides the interaction with SDH, GPX-4, and 5α-RD. Therefore, GFSV-loaded ERLN cargoes are a promising strategy for PCA treatment. *In vivo* studies are necessary to confirm these findings for clinical applications.

## Introduction

Prostatic cancer (PCA) is the second most frequently identified cancer and the fifth leading reason of cancer death among men globally [1,2] Aging, race, unbalanced diets, smoking, alcohol addiction, and family history are the major risk factors for PCA. Moreover, unhealthy lifestyles, obesity, and diabetes are established risk factors for PCA [1]. The prevalence and death of PCA differ significantly among different countries and regions [2]. The aggressive forms of PCA can manifest rapid growth and metastasize to adjacent organs [2]. The prediction of PCA provides a higher chance of cure [2]. Oxidative stress and androgens activate genetic and epigenetic factors that mediate malignant transformation and the development of PCA [3]. Tyrosine kinase inhibitors (TKIs) were described as therapeutic agents for PCA management [4]. TKIs are inhibitors that interfere with multiple kinases in uncontrolled cell growth signals [4]. Despite the extensive medical applications of TKIs, their frequent use induces various organ dysfunction, multidrug resistance (MDR), and failure of therapeutic influence [5]. MDR of cancer to TKIs attributed to epigenetic effect, gene mutations, and irregular drug metabolism [6]. Gefitinib (GF) is a member of the TKIs family that showed multiple systemic toxicities due to high dosing and MDR [7].

Likewise, the targeting of lipids is an innovative therapeutic strategy for PCA therapies, yet, abnormal lipid metabolism is associated with the malignant transformation of prostatic cells [8]. In this regard, ample studies documented that statin drugs for PCA control [9]. However, statins as mono and combined therapy could increase the drug sensitivity of PCA [8,9]. Thus, statin therapy is suggested to improve the prognosis of PCA patients [10]. In this context, the use of statin in combination therapy reduced the risk of a PCA patient’s mortality [11].

Moreover, several studies documented a positive correlation between statins use and decreased risk of PCA [12]. The anticancer mechanism of statins is mediated by the diminution of cholesterol, and steroid hormone production [13]. Furthermore, statins lower coenzyme-Q-10 production, lower ATP, and decrease G-protein prenylation [14]. Interestingly, statins induced cytotoxicity without affecting DNA function. Accordingly, simvastatin (SV) and other members of statins are repurposed as anticancer agents [15]. Further studies are still required to confirm this issue.

Nanodelivery is an exceptional technique to treat several diseases including PCA. Specifically, the surface-engineered nano-delivery systems could induce selective and simultaneous delivery of the drugs to PCA with negligible adverse effects [16]. Besides, nano-delivery cargoes could overcome biological barriers, and mediate active, and passive drug targeting to the tumor cells [17]. Responsive nano-delivery systems are recommended for targeting numerous diseases such as PCA and others [15]. However, PCA exhibits MDR to chemotherapy, radiation, and androgen deprivation therapy [16]. Thus, responsive nano-delivery systems such as photodynamic responsive therapy, photothermal responsive therapy, physical responsive therapy chemical responsive therapy, and enzymes responsive therapy are suggested as a potential therapy for PCA [16].

Enzymes responsive therapy is promising for drug-targeting into cancer cells, however, tumor cells overexpress enzymes such as esterases, lipases, phospholipases, proteinases, and other enzymes to promote cancer growth, progression, and metastasis. Specifically, esterase is overexpressed in several folds in cancer cells [17]. Consequently, the substrate of esterase can be used as a tool for the fabrication of cancer-selective nano-delivery systems [18]. Likewise, phospholipases and other enzyme-triggered drug cargoes were documented for cancer drug targeting [19]. For instance, esterase-sensitive drug carriers are hydrolyzed by intracellular esterase to release oncotherapy in cancer cells in a selective manner [17].

Lipid nanoparticles (LNs) such as nanostructured lipid carriers (NLCs) and others are biocompatible, biodegradable, and non-immunogenic. LNs have high drug loading, stability, sustained drug release, and easy engineering [20]. Furthermore, LNs enhance drug solubility and permeability across biomembranes [20]. Specifically, the surface of LNs can be modified by ample ligands that can bind with the receptors on cancer cells [20,21]. The lipophilic drugs are impeded in the nonpolar core of LNs to facilitate solubility and cell permeability [20]. Despite, the translation of LNs into clinical applications, they are still challenging issues [22], thus, coating agents could resolve the limitation and delivery challenges of LNs [20]. Accordingly, polymers, proteins, lipids, and phospholipids are harnessed for coating and design of enzyme-responsive nano-delivery systems [23]. In this context, phospholipids decoration of LNs induced biocompatibility, stability, and drug release pattern [23,24]. Moreover, the presence of lipids and phospholipids could mediate selective tumor targeting, however, tumor cells such as PCA overexpress phospholipases that trigger the drug release in the tumor cells [23,24]. In this regard, phospholipase-triggered liposomes were demonstrated for targeting chemotherapeutic agents [23]. Subsequently, developing esterase-triggered nanoparticles could mediate the selective accumulation of anticancer medicines at the tumor site [17]. Even though esterase-responsive nanoparticles were documented for drug delivery into PCA cells [25], further studies are required to address this issue.

Frequently chitosan (CS) is used for the surface engineering of nanoparticles, CS is a natural polymer that has biocompatibility, and biodegradability [26]. The surface engineering of NLCs by CS-induced positive zeta potential enhances cellular penetration and improves the bioavailability and efficacy of the drugs [27]. CS-engineering of LNs promotes drug absorption by prolonging the contact time with the cell membrane [28]. CS-induced positive charge assists electrostatic attraction of nano-delivery systems to the biomembranes due to the anionic characteristic of the cell membrane [28].

Together, GF and SV therapy requires high dosing due to lipophilicity, low solubility low bioavailability, none-selective distribution, and MDR. These effects worsen the unwanted systemic impact of these drugs. Therefore, this study aims to develop enzymes-response LNs (ERLNs) loaded with GF and SV (ELRNGFSV) to target PCA. ELRNGFSV cargos were prepared using down-top, followed by top-down technique, next, they were shielded by phospholipids, triacylglycerol, and CS to form a triple corona on ERLNs. Afterward, the prepared cargoes are characterized in terms of particle size (PS), zeta potential (ZP), and polydispersity index (PDI). The biosafety and cytotoxicity of ERLN preparations were studied using erythrocyte suspension and PC-3 cell line as surrogate models for PCA. The Merck Molecular Force Field 94x (MMFF94x) and the semi-empirical Austin Model 1 (AM1) allow analysis of the interactions between the drug and target enzymes. Recognizing additional potential targets of GF and SV enhanced the discovery of a therapeutic strategy for PCA. Therefore, the impact of GF and SV on succinate dehydrogenase (SDH), glutathione peroxidase-4 (GPX-4), and 5α-reductase (5α-RD) was studied using MDS.

## Materials and Methods

### Materials

Gefitinib (GF) was obtained from Beijing Mesochem Technology Co. Ltd. (184475-35-2, Beijing, China). Simvastatin (SV) was supplied as a gift from Riyadh Pharma Company (79902-63-9, Riyadh, Saudi Arabia). Stearic acid (SA) SA was obtained from BDH (57-11-4, Poole, UK). Oleic acid (OA) was acquired from Avonchem (112-80-1, Cheshire, UK). Chitosan (CS) low molecular weight abstained from Sigma-Aldrich (448869, St. Louis, MO, USA), and Pluronic F-68 were obtained from Sigma Aldrich (P7061, St. Louis, MO, USA). All of the remaining chemicals are available of high analytical grade.

### Assembly of ELNGFSV

Down-top, top-down ultrasonic melt-emulsification scheme was employed to gather plain enzyme-responsive lipid nanoparticles (PERLN), ERLNGFSV, and CS-engineered PERLN (CPERLN) and ERLNGFSV (CERLNGFSV) [28,29]. The components of the preparations are indicated in Table 1. Firstly, the precise amount of SA as solid lipid, OA as liquid lipid, lipoid-S100, coconut oil, GF, and SV were weighed and placed in a beaker to make the lipid phase. In another beaker, Pluronic F-68 as surfactant and distilled water were placed to prepare the aqueous phase. The pluronic F-68 was left at 4°C for a complete solution. Next, lipid and aqueous phases were concurrently heated using a metal bath (Hot plate) to 80°C. Then, both hot aqueous and lipid phases were mixed and stirred together to prepare the primary micro-emulsion. Finally, ERLNGFSV was attained after sonication of the primary micro-emulsion using a probe sonicator (ATP-150, Mumbai, India) at 80% voltage efficiency for 6 cycles. Each cycle extended for 40 s disrupted with a resting period extended for 5 s. The consistent milky appearance of the preparations was used as an indicator for LN production. Coconut oil and lipoid-S100 were used to form an esterase-responsive shell on the LNs to produce ERLN. Coconut oil contains short-chain triacylglycerol, however, lipoid-S100 contains phosphatidylcholine. After preparations, the assembled ERLNGFSV were kept in a refrigerator (4°C) for further use.

**Table 1:** The composition of PERLN, ERLNGFSV, CPERLN, and CERLNGFSV

Ingredients	Role	PERLN	ELRNGFSV	CPERLN	CERLNGFSV
Stearic acid (mg)	Solid lipid	900	900	900	900
Oleic acid (mg)	Liquid lipid	200	200	200	200
Coconut oil (mg)	Esterase substrate	400	400	400	400
Lipoid S-100 (mg)	Phospholipase substrate	40	40	40	40
Simvastatin (μg)	Active ingredient	–	50	–	50
Gefitinib (μg)	Active ingredient	–	50	–	50
Pluronic F-68 (mg)	Surfactant	500	500	500	500
Distilled water (mL)	Solvent	48	48	48	48
Chitosan 5% (mL)	Coating agent	–	–	Equal volume	Equal volume

### CS engineering of ELNGFSV

The CS engineering of ERLNGFSV produces CERLNGFSV accomplished by mixing equal amounts of ERLNGFSV, and CS solution in acetic acid. The CS 1% was dissolving in 0.5% acetic acid solution. The CS solution was added dropwise to ELNGFSV under magnetic stirring (C-MAG HS 10, Deutschland, Germany) for 20 min to produce CERLNGFSV. The prepared formulations were continuously stirred for 2 h [28,29]. The obtained CERLNGFSV preparations were kept in a refrigerator (4°C) for further use.

### Characterization of ERLNGFSV

PS, PDI, and ZP of prepared PERLN, ERLNGFSV, CPERLN, and CERLNGFSV were categorized using a Zetasizer Nano ZS (Malvern Instruments, UK). Each PERLN, ERLNGFSV, CPERLN, or CERLNGFSV was diluted (1:1000) in phosphate-buffered saline and assessed at 25°C. Each sample was measured in triplicate.

### The particle morphology

The selected PERLN, ERLNGFSV, CPERLN, and CERLNGFSV were subjected to surface morphology and visualized by scanning electron microscopy (JSM-6360 LV, JEOL, Tokyo, Japan). The samples were diluted with ultra-pure water at dilution 2%, dried by lyophilization using (Alpha 1-4 LD Plus, Martin Christ Gefriertrocknugs Anlagen GmbH, Osterode am Harz, Germany), and fixed on carbon tape and sputter-coated with a thin gold layer under an argon atmosphere using a gold sputter module in a high-vacuum evaporator (JFC-1100 fine coat ion sputter; JEOL). Finally, the samples were then scanned by scanning electron microscopy (JEOL JSM-7610F, (Tokyo, Japan). The photomicrographs were taken at an acceleration voltage of 20 kV.

### Biocompatibility studies

To investigate the biocompatibility of the prepared ERLN, 2 mL of blood were collected from a healthy male volunteer aged 53 years, who do not suffer from acute or chronic illness, after verbal agreement and informed consent. The red blood cells (RBCs) were utilized to investigate the hemocompatibility of the preparations as described by the previous study [30]. The RBCs were separated and diluted with a physiological saline solution to obtain 2% erythrocyte suspension. To achieve the biocompatibility studies, the RBCs treated with the solution of DMSO, GF, SV, as well as PERLN, ERLNGFSV, CPERLN, and CERLNGFSV and compared to positive and negative control. Group 1: The negative control doesn’t elicit a hemolytic effect on RBCs, the RBCs were treated with isotonic phosphate-buffered saline. Group 2: positive control elicits complete RBC hemolysis; the RBCs were incubated with distilled water to induce hypotonic lysis. Group 3: The RBCs were incubated with 1% DMSO as the vehicles incubated group. Group 4: The RBCs were incubated with GF dissolved in 1% DMSO. Group 5: The RBCs were incubated with SV dissolved in 1% DMSO. Group 6: The RBCs were incubated with GF plus SV dissolved in 1% DMSO. Group 7: The RBCs were incubated with PERLN. Group 8: The erythrocytes were incubated with ERLNGFSV. Group 9: The blood suspension RBCs were incubated with CPERLN. Group 10: The erythrocytes were incubated with CERLNGFSV.

The incubated RBCs in all groups were mixed by gentle inversion several times kept at 37°C for 1 h. Afterward, the samples were centrifugated at 3000 rpm for 5 min using Centrifuge EBA 20 Hettich, Germany. Afterward, the absorbance of the clear supernatant was measured at 570 nm using UV-Vis Spectrophotometer (Genesys 10S UV-VIS, Thermo Scientific, Waltham, MA, USA). The hemolysis percentage was calculated using the following equation:

$$\begin{array}{l} Hemolysis=\\ \frac{Absorbance\;sample-absorbance\;negative\;control}{\;absorbance\;positive\;control-absorbance\;negative\;control}*100 \end{array}$$

### PC-3 cell death studies

In this study, the PC-3 cell line was used as a surrogated model for PCA, accordingly, the PC-3 cell line was cultivated in a Dulbecco’s Modified Eagle’s Medium (DMEM, Gibco, Grand, 21063029, Island, NY, USA) DMEM containing 10% fetal bovine serum (FBS, 11I249; Shanghai, China), 1% v/v penicillin-streptomycin (Thermo Fisher Scientific, 15140148, Waltham, MA, USA) and incubated in a 95% air-humidified atmosphere containing 5% CO_2_ at 37°C in sterile flasks. In the case of our current study involving the PC 3 cell line, we have not observed any signs of contamination. PC-3 cells were suspended in a culture medium and seeded in two different 96-well microtiter plates in triplicate at a density of 5 × 10^3^ cells/well for 24 h. The cultivated PC-3 cell line was treated with free GF, SV, and their combination as well as PERLN, ERLNGFSV, CPERLN, and CERLNGFSV as follows. Group 1: The PC-3 cell line was treated with 1% DMSO as a control group. Group 2: The PC-3 cell line was treated with GF in dissolved 1% DMSO. Group 3: The PC-3 cell line was treated with SV dissolved in 1% DMSO. Group 4: PC-3 cells were treated with a combination of SV and GF dissolved in 1% DMSO. Group 5: The PC-3 cell line was treated with PERRLN. Group 6: The PC-3 cell line was treated with ERLNGFSV. Group 7: The PC-3 cell line was treated with CPERLN. Group 8: The PC-3 cell line was treated with CERLNGFSV. The GF and SV doses were selected based on the previously published work [31]. All groups were incubated for 24, 48, and 72 h. The percent of cell viability was detected using MTT (3-(4, 5-dimethylthiazolyl-2)-2, 5-diphenyltetrazolium bromide) assay. In the MTT assay, the mitochondrial enzyme succinate dehydrogenase (SDH) of viable cells converts hydrophilic tetrazolium salt (yellow color) into a hydrophobic purple dye known as formazan, and the intensity of the color indicated to the percent viable cells.

A 50 µL aliquot of MTT (0.42 mg/mL) was added to each well, and the microplate was returned to the incubator for 4 h. After incubation, the media was removed, the MTT was solubilized in 1 mL of isopropyl alcohol, and the absorbance of each sample was measured at 570 nm using a microplate reader (ELX 800; Bio-Tek Instruments, Winooski, VT, USA).

$$Viability\;\left(\%\right)=\frac{optical\;density\;of\;treated\;group}{\;optical\;density\;of\;untreated\;group}*100$$

### Study of PC-3 apoptosis using flow cytometry

PC-3 apoptosis was examined using an annexin V-fluorescein isothiocyanate (FITC)/propidium iodide apoptosis detection kit (BioLegend, 640932, CA, USA). Firstly, PC-3 cells were seeded into 12-well plates at a density of 5 × 10^4^ cells/well in 1 mL DMEM for 12 h. Next, PC-3 cells were treated with 1% DMSO (Control), GF, SV, GF plus SV, PERLN, ERLNGFSV, CPERLN, and CERLNGFSV. Then, the cells were incubated for 24 h, afterward, they were washed with phosphate-buffered saline and lysed (0.25% trypsin, for 7 min at 37°C) with trypsin. Then, the cells were harvested by centrifugation at 2000× *g*. Subsequently, cells were diluted by binding buffer with double distilled water, and 500 μL of suspension cells with 1 × binding buffer were prepared. After that, 500 μL of control and the treated cell was incubated with 5 μL of Annexin V-FITC and 10 μL of propidium iodide at room temperature for 30 min in darkness. Finally, the fluorescence of the cells directly was determined using a Cytomics FC500 flow cytometer, (Beckman Coulter, CA, USA).

### Molecular docking study

This study employed molecular modeling techniques to investigate the interactions of GF and Simvastatin acid (Tenivastatin) a metabolite of SV with three key targets: SDH, GPX-4, and 5α-RD. Molecular modeling analyses were carried out with Molecular Operating Environment (MOE) 2015.10 (https://www.chemcomp.com/) (accessed on 03 November 2024), the software (Chemical Computing Group Inc., Montreal, QC, Canada). This study aimed to gain a deeper understanding of how these drugs interact with these enzymes, ultimately contributing to the development of novel therapeutic agents.

### Preparation of ligand structures

The molecular structures of two compounds, GF and Tenivastatin, were acquired in the structure-data file (.sdf) format from the PubChem database (https://pubchem.ncbi.nlm.nih.gov/substance/?source=chemidplus&sourceid=0121009776#section=2D-Structure, https://pubchem.ncbi.nlm.nih.gov/compound/Gefitinib#section=2D-Structure, accessed on 03 November 2024). These structures were then imported into the MOE database. Using the builder utility in the MOE software, the compounds were systematically prepared as follows: The compounds were prepared for analysis by protonation at pH 7, adding explicit hydrogens, generating 3D coordinates, assigning partial charges, and minimizing their energy using the MMFF94x: EHT force field until a root-mean-square (RMS) gradient of 0.01 kcal/mol/Å^2^ was reached.

The MMFF94x: EHT force field is designed for small organic molecules in medicinal chemistry. It is a modified version of MMFF94s, with the key difference being that it forces conjugated nitrogen to be planar. It is an all-atom force field that does not use lone pairs and is compatible with the Generalized Born solvation model [32]. MOE offers additional methods for calculating partial charges, including the Gasteiger (PEOE) (Partial Equalization of Orbital Electronegativities) formalism, which is an iterative method where charge is transferred between bonded atoms until equilibrium is reached, AM1-derived charges, and Electrostatic Potential charges calculated using the MOPAC program. MOPAC charges are derived from semi-empirical calculations but may require long computation times and are limited to very small molecules with fewer than 60 heavy atoms [33].

### Preparation of enzyme structures

The crystalline models of four distinct enzymes were obtained from X-ray diffraction data, with the following specifications: SDH (PDB code 2FBW) at a resolution of 2.06 Å, R-Value Free of 0.215, R-Value Work of 0.171, and R-Value Observed of 0.173. GPX-4 (PDB code 2OBI) at a resolution of 1.55 Å, R-Value Free of 0.186, R-Value Work of 0.164, and R-Value Observed of 0.165. 5α-Reductase (5α-RD) (PDB code 3G1R) at a resolution of 1.70 Å, R-Value Free of 0.205, R-Value Work of 0.175, and R-Value Observed of 0.177.

The preliminary step in the structural refinement involved the utilization of the MOE Structure. Preparation tool to amend any inaccuracies in the original crystal structures of the selected enzymes [34]. This correction included the allocation of hydrogen atoms according to the prescribed norms. All water molecules and cofactors present in the target proteins (PDB codes 2FBW, 2OBI, and 3G1R) were removed. Afterward, the Gasteiger method was employed to ascertain the partial charges [35]. The active sites were delineated based on the proximity of residues—specifically, those within a 10 Å radius from the inhibitor bound in the crystal structure, forming a collective region encompassing all connected ligands. This step involved evaluating every atom located within 10 Å of any part of the ligand to ensure comprehensive consideration of potential interaction [36].

### Docking experiment

MDS were carried out by aligning the energetically optimized compounds with their respective co-crystallized counterparts found within the Protein Data Bank (PDB) entries for the four target enzymes. Once the alignment was established, the original co-crystallized ligands were excised from the PDB structures [37]. The positioning of the ligands during the docking procedure was achieved through the integrated Triangle Matcher algorithm provided by the chosen docking software. Subsequently, to evaluate and prioritize the possible binding conformations—commonly known as poses—an estimation of the free energy of ligand binding was calculated using the GBVI/WSA dG scoring function [38]. This scoring function provides a numerical value indicative of the binding affinity between the enzyme and the ligand. The pose that exhibited the lowest S-core, suggesting the highest binding affinity within the applied model, was earmarked for additional examination and interpretation.

### Statistical analysis

Data analysis was achieved by GraphPad software, version 5 (GraphPad, ISI Software Inc., La Jolla, CA, USA). The results were compared using a one-way analysis of variance. Data were expressed as mean ± SD and *p*-value < 0.05 were used as criteria for significance.

## Results

### Influence of ERLN composition

In the present results, PERLN, ERLNGFSV, CPERLN, and CERLNGFSV were organized using successive down-top, top-down ultrasonic melt-emulsification techniques. The molecules of lipids and aqueous phases were gathered in microemulsion, and upon sonication, they were converted into ERLNs. Table 1 displays the composition of ERLN.

**Figure 1:**
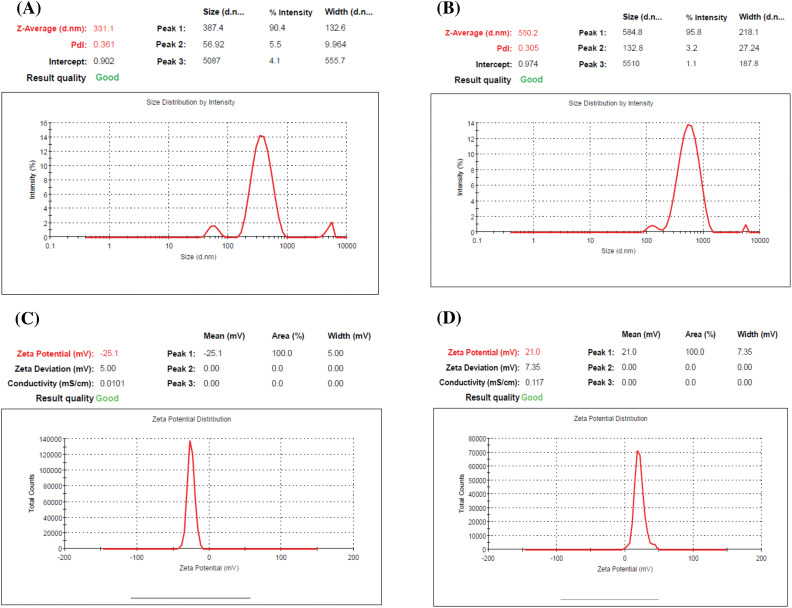
The particle size distribution and zeta potential graph of select ERLN, (A) PELRN, (B) ERLNGFSV, (C) ELRNGFSV, (D) CERLNGFSV.

The present results revealed that the prepared ERLN has a nanoscale size range (353–449 nm), see Fig. 1A,B. Moreover, ERLN cargoes are homogenous as confirmed by low PDI (0.198–0.321), see Table 2. The current study indicated the ZP of PERLN, and ERLNGFSV was negative values (−25.00 to −27.00 mV), see Fig. 1C,D. While CS capping shifted the ZP from positive values (22.00 to 21.00 mV) in the case of CPERLN and CERLNGFSV, see Fig. 1D and Table 2.

**Table 2:** The particle size, zeta potential, polydispersity index, and drug content of PERLN, ERLNGFSV, CPERLN, and CERLNGFSV

	PERLN	ERLNGFSV	CPERLN	CERLNGFSV
Particle size (nm)	353.00 ± 62.00	377.00 ± 73.00	410.00 ± 83.00	449.00 ± 88.00
Zeta potential (mV)	−25.00 ± (−3.00)	−27.00 ± (−2.00)	22.00 ± 2.00	21.00 ± 3.00
Polydispersity index	0.198 ± 0.034	0.321 ± 0.040	0.220 ± 0.020	0.288 ± 0.100
GF+SV (mg/mL)	–	50 + 50	–	50 + 50

Note: Data were expressed as the mean ± SD, Sample number, N = 3.

**Figure 2:**
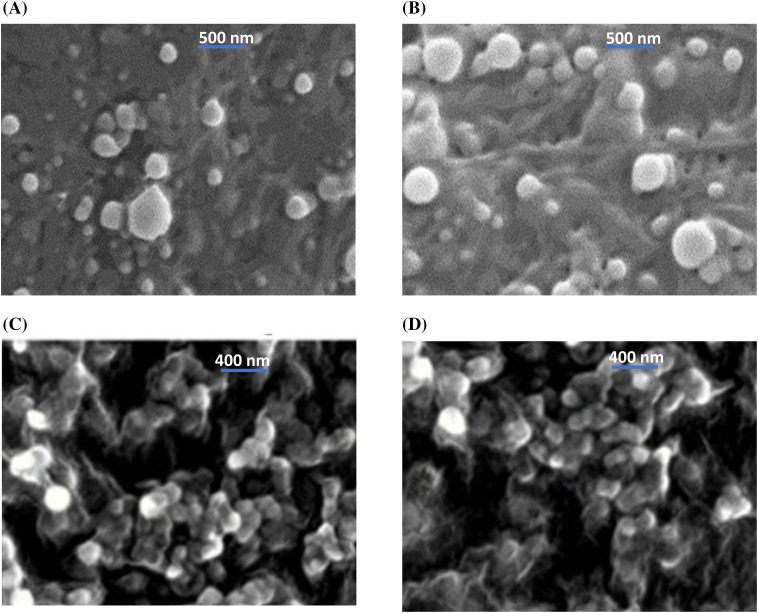
Scanning electron micrography of the selected ERLN formulation, (A) PELRN, (B) ERLNGFSV, (C) CPELRN, (D) CRLNGFSV.

SEM imaging revealed that the shape of both PERLN and ERLNGFSV appeared in spherical and oval shapes with the presence of some aggregates (Fig. 2A,B). In contrast, the CERLN and CERLNGFSV have a circular appearance and are adhered to each other’s (Fig. 2C,D).

**Figure 3:**
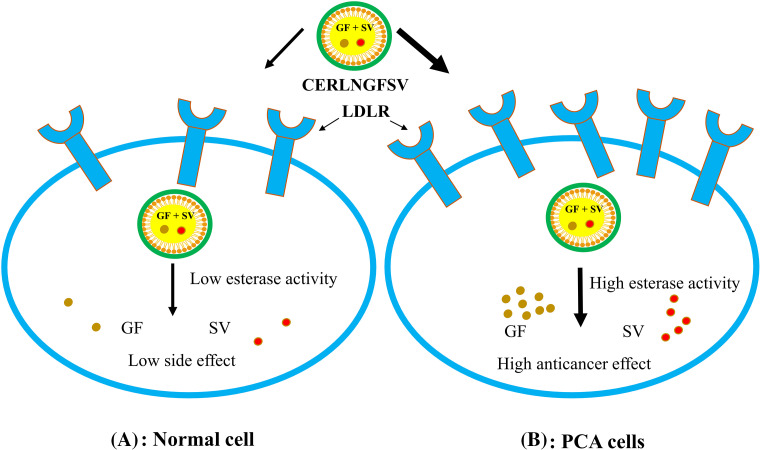
The cellular uptake of ERNLGFSV by low-density lipoprotein receptors (LDLR) overexpressed on PCA cell surfaces.

In this study, CS addition achieved the formation of a cationic cap on the ERLN surface. ERLN mediates active targeting of GF and SV into PC-3 by receptors endocytosis machinery due to the nanoscale character of ERLN cargoes, see Fig. 3. Thus, both GF and SV are less introduced into normal cells, compared to PC-3 cells that overexpressed low-density lipoprotein receptors (LDLR). In the present study, the surface of ERLN was engineered by triacylglycerol, and phosphatidylcholine as substrates for hydrolytic enzymes. In the intracellular milieu, ERLN cargoes can release their GF and SV payload under the effect of lipase, and phospholipase enzymes overexpressed in PC-3 [22,26]. The current findings are aligned with several studies that documented the higher cellular uptake of LNs loaded with chemotherapeutic drugs. However, LNs have a high affinity for LDLR and fatty acid receptors that are overexpressed on the surface of cancer cells [22,26]. Moreover, LNs have s native tropism to the lymphatic system to fight hidden cancer cells [36].

### Biocompatibility of ERLNGFSV

**Figure 4:**
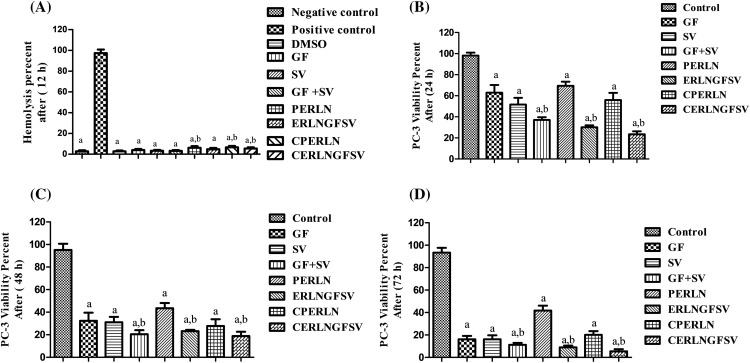
Effect of the prepared ERLNs on biocompatibilityand viability of PC-3 cell line. (A) Biocompatibility is indicated by the effect of different treatments on the percent of red blood cell hemolysis. The negative control doesn’t elicit a hemolytic effect on RBCs, positive control elicits complete RBCs hemolysis. DMSO is a solvent for GF and SV. a: significant decrease from positive control (complete hemolysis). b: significant increase from negative control (no hemolysis). (B) PC-3 cell line viability percent using MTT assay after different treatment at 24 h. (C) PC-3 cell line viability percent using MTT assay after different treatment at 48 h. (D) PC-3 cell line viability percent using MTT assay after different treatment at 72 h. a: significant decrease from 1% DMSO treated (control) PC-3 cells. b: significant decrease from GF or SV PC-3 treated groups. Sample number, N = 6.

In the current study, the hemolysis percent of DMSO, GF, SV, and GFSV combination, as well as PERLN, ERLNGFSV, CPERLN, and CERLNGFSV was less than 10 percent, after 24 h (Fig. 4A).

### Effect of ERLN on PC-3 cell viability

The present results showed that formazan development by PC-3 cells was decreased upon incubation with GF, SV, and GFSV in DMSO, PERLN, ERLNGFSV, CPERLN, and CERLNGFSV compared to the control group untreated cells. Interestingly, the viability percent of PC-3 was decreased by the time upon treatment with SV+GF, and ERLNGFSV. Furthermore, CERLNGFSV augments cell death compared with other treated groups Fig. 4B–D demonstrate the effect of different treatments on PC-3 cell viability after 24, 48, and 72 h incubation time, respectively. Moreover, these findings confirmed the synergistic effect of GF plus SV codelivery on PC-3 death compared to control, free GF, and SV. Additionally, ERLNGFSV and CERLNGFSV induced an increase in PC-3 cell death increases by increasing the incubation time.

### Effect of ERLN on PC-3 apoptosis

**Figure 5:**
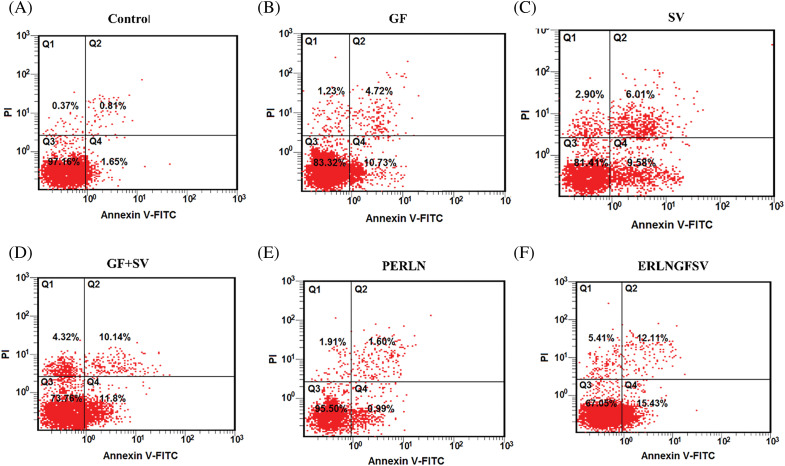

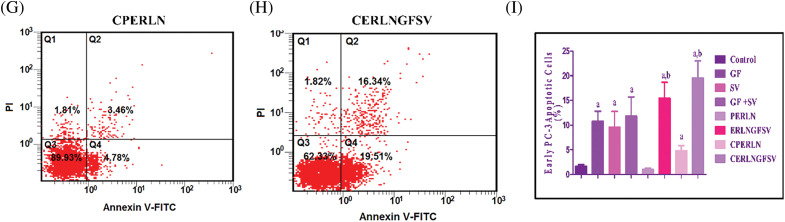
Representative flow cytometry images of Annexin V-FITC/propidium iodide double-staining and the percent of PC-3 apoptotic cells upon exposure to different treatments determined by flow cytometric analysis. (A) DMSO, (B) GF, (C) SV, (D) GF+SV, (E) PERLN, (F) ERLNGFSV, (G) CPERLN, and (H) CERLNGFSV. (I) Effect of the different on the percent of early PC-3 apoptotic cells compared to control cells. a: significant increase from control PC-3 cells. b: significant increase from GF or SV PC-3 treated cells. 6 samples per group.

In the present results, the treatment of PC-3 cells with DMSO, GF, SV, GF+SV, PERLN, ERLNGFSV, CPERLN, and CERLNGFSV induced apoptotic of PC-3 cells compared to control PC-3 cells. The percentage of apoptotic cells was increased upon exposure to pure drugs GF, SV, GF+SV, ERLNGFSV, and CERLNGFSV, respectively. Treatment of PC-3 cells with GF plus SV, ERLNGFSV, and CERLNGFSV showed a marked percentage of apoptotic cells compared to other groups, Fig. 5A–H indicated cytometry images of Annexin V-FITC/propidium iodide double-staining and the percent of PC-3 apoptotic cell upon treatment GF, SV, and GF+SV, PERLN, ERLNGFSV, CPERLN, and CERLNGFSV compared to control cells. Fig. 5 displays the effect of different treatments on the percent of early PC-3 apoptotic cells compared to control cells.

### Molecular docking

#### SDH

To identify currently approved drugs that could potentially bind to the interacting surfaces of SDH, GPX-4, and 5αRD, GF and Tenivastatin were docked against these binding sites using the rigid-receptor model in MOE 2015.01. This model captures the protein conformations, providing a high enrichment of docked compound poses. The “Triangle Matcher,” suitable for standard and well-defined binding sites, was used as the ligand placement method. The initial London free energy of binding for 30 poses was rescored using the generalized Born volume integral (GBVI) model to obtain the final S score for five poses. A higher negative S score indicates better ligand interaction with the protein and a more stable ligand-protein complex. The chemical structures and estimated free energy of binding (S score) of the top poses of the compounds are presented in Table 3. The top-ranking compounds based on the predicted S score were mostly peptide-based drugs (approved, investigational, and experimental drugs).

**Table 3:** Summary of the type of interaction, interatomic distances, interaction energies, and docking scores for Gefitinib and Tenivastatin with Succinate Dehydrogenase (SDH, PDB: 2FBW)

Tested molecule	Ligand	Receptor	Interaction	Distance	E (kcal/mol)	Docking score (kcal/mol)
Gefitinib	C28	O ILE27 (C)	H-donor	3.51	−0.3	−9.1956
	C32	SD MET36 (C)	H-donor	3.57	−0.6	
	6-ring	CD1 ILE218 (B)	pi-H	4.21	−0.3	
	6-ring	CD1 ILE40 (C)	pi-H	3.66	−0.3	
Tenivastatin	O2	NE1 TRP173 (B)	H-acceptor	3.08	−2	−8.0977
	O2	OH TYR58 (D)	H-acceptor	2.97	−1.6	
	O5	CA ILE40 (C)	H-acceptor	3.29	−0.3	
	O7	NE2 HIS216 (B)	Ionic	3.94	−0.6	
	O7	NE ARG43 (C)	Ionic	3.63	−1.4	
	O7	NH1 ARG43 (C)	Ionic	3.75	−1.1	
	O7	NH2 ARG43 (C)	Ionic	2.99	−4.5	

**Figure 6:**
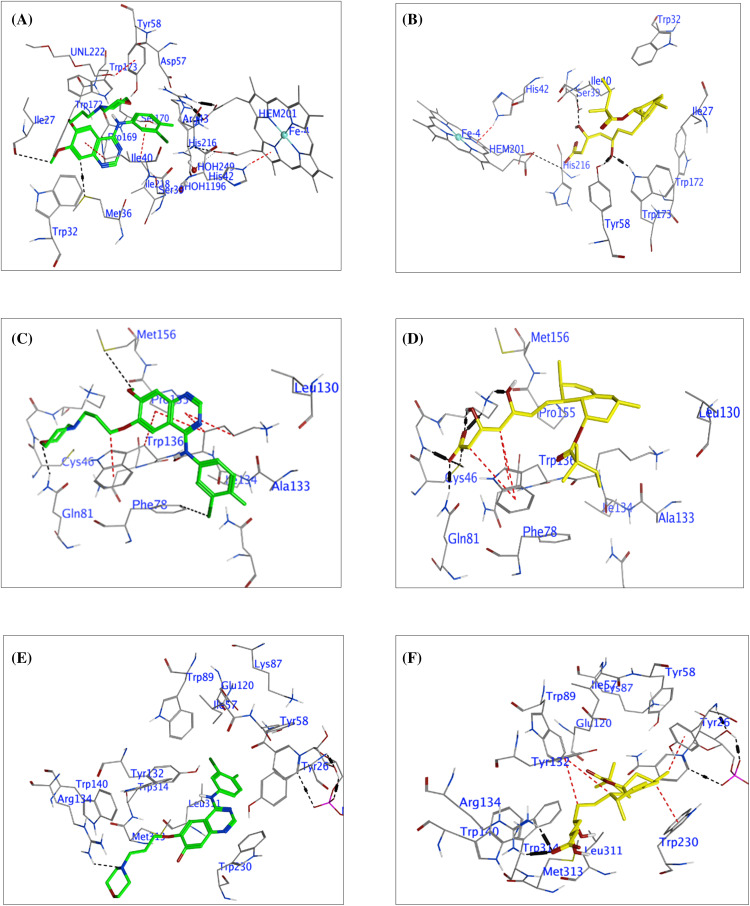
The molecular docking interactions between Succinate Dehydrogenase (SDH), Glutathione Peroxidase-4 (GPX-4), 5α-Reductase (5α-RD), and the compounds Gefitinib (green) and Tenivastatin (yellow). SDH interaction (A) Gefitinib, (B) Tenivastatin. GPX-4 interaction, (C) Gefitinib, (D) Tenivastatin. 5α-RD interaction, (E) Gefitinib, (F) Tenivastatin.

Specifically, for GF, interactions included a hydrogen-donor interaction between the ligand atom C28 and the backbone oxygen of ILE27 (C) at a distance of 3.51 Å, with an interaction energy of −0.3 kcal/mol. Another hydrogen-donor interaction involved ligand atom C32 and the sulfur atom of SD in MET36 (C), with a distance of 3.57 Å and an interaction energy of −0.6 kcal/mol. Hydrophobic pi-H interactions were evident between the 6-ring of GF and the delta carbon of ILE218 (B) and ILE40 (C), at distances of 4.21 and 3.66 Å, each with interaction energies of −0.3 kcal/mol. GF docking score was computed to be −9.1956 kcal/mol. Fig. 6A,B is the summary of molecular docking interactions of GF (green) and SVA (yellow) with the active site residues of (SDH, PDB: 2FBW).

For SVA, various interactions were identified: A hydrogen-acceptor interaction with O2 of SVA and NE1 of TRP173 (B) at a distance of 3.08 Å and an interaction energy of −2 kcal/mol. Another hydrogen-acceptor interaction was observed with O2 of SVA and the hydroxyl group of TYR58 (D), with a distance of 2.97 Å and energy of −1.6 kcal/mol. Additional hydrogen-acceptor interactions were found between O5 of SVA and CA of ILE40 (C) at a distance of 3.29 Å and energy of −0.3 kcal/mol. Ionic interactions were noted with O7 of SVA and NE2 of HIS216 (B), NE, NH1, and NH2 of ARG43 (C), with distances ranging from 2.99 to 3.94 Å and interaction energies ranging from −0.6 to −4.5 kcal/mol. The docking score of SVA was calculated to be −8.0977 kcal/mol.

MDS revealed that both GF and SVA interact with SDH at its active site, primarily through hydrogen bonding and hydrophobic interactions. The presence of multiple interactions and their distance from the active site residues suggests that both compounds have the potential to inhibit the enzymatic activity of SDH through different binding modes. GF presents hydrogen-donor interactions and pi-H interactions with amino acid residues within the active site, indicating a possible significant interaction with the receptor. Despite the relatively low individual interaction energies, the overall docking score of −9.1956 kcal/mol suggests GF is a potent inhibitor.

SVA shows a variety of interaction types, including hydrogen-acceptor and ionic interactions, with lower distances to the active site residues. The strongest interaction appears to be an ionic bond between oxygen O7 of SVA and NH2 of ARG43 (C) with an interaction energy of −4.5 kcal/mol. This interaction, along with others, contributes to the favorable docking score of −8.0977 kcal/mol, suggesting that SVA is also a potential inhibitor of SDH.

#### GPX-4

In this molecular docking study, the interactions between GPX-4 and two ligands, GF and SVA, were investigated in Table 4. GF demonstrated hydrogen bonding with the catalytic triad residues C46 and Q81, along with pi-stacking interactions with W136. On the other hand, SVA exhibited multiple hydrogen bonding interactions with CYS 46 and LYS 48, including an ionic interaction with LYS 48. Both ligands displayed favorable interactions with the critical residues involved in GPX4’s enzymatic activity. Notably, SVA ionic interaction may contribute to its potentially stronger binding affinity compared to GF.

**Table 4:** The type of interaction, interatomic distances, interaction energies, and docking scores for Gefitinib and Tenivastatin with the active site residues of Glutathione Peroxidase-4 (GPx4, PDB: 2OBI)

Tested molecule	Ligand	Receptor	Interaction	Distance	E (kcal/mol)	Docking score (kcal/mol)
Gefitinib	C28	SD MET156 (A)	H-donor	4.39	−0.2	−6.5444
	N43	O LYS135 (A)	H-donor	3.03	−0.5	
	O7	NE2 GLN81 (A)	H-acceptor	3.44	−0.4	
	CL49	CE1 PHE78 (A)	H-acceptor	3.85	−0.2	
	C21	6-ring TRP136 (A)	H-pi	3.6	−0.5	
	6-ring	CB LYS135 (A)	pi-H	4.66	−0.4	
	6-ring	CB LYS135 (A)	pi-H	4.06	−0.5	
	6-ring	CD LYS135 (A)	pi-H	4.41	−0.2	
	6-ring	CB TRP136 (A)	pi-H	4.79	−0.2	
Tenivastatin	O2	SD MET156 (A)	H-donor	4.14	−0.2	−7.1429
	O7	SG CYS46 (A)	H-donor	3.37	−0.2	
	O8	SG CYS46 (A)	H-donor	3.53	−0.7	
	O2	NZ LYS48 (A)	H-acceptor	3.09	−3.3	
	O7	SG CYS46 (A)	H-acceptor	3.37	−0.5	
	O7	N GLY47 (A)	H-acceptor	3.14	−4.5	
	O7	NE2 GLN81 (A)	H-acceptor	3.1	−2.2	
	O8	NZ LYS48 (A)	H-acceptor	2.82	−15.6	
	O8	NZ LYS48 (A)	Ionic	2.82	−5.8	
	C46	5-ring TRP136 (A)	H-pi	3.7	−0.2	
	C46	6-ring TRP136 (A)	H-pi	4.59	−0.2	
	C63	6-ring TRP136 (A)	H-pi	4.81	−0.2	

These findings offer valuable insights into the molecular mechanisms underlying the inhibitory activity of these ligands against GPX-4, although experimental validation is necessary to corroborate these predictions. Table 4 includes the type of interaction, interatomic distances, interaction energies, and docking scores for each compound with GPX-4 (GPx4, PDB: 2OBI). Fig. 6C,D is a summary of molecular docking interactions of GF and SVA with the active site residues of GPX-4 (GPx4, PDB: 2OBI).

The docking results provide insights computational into the potential inhibitory effects of GF and SVA against GPX-4 based on their interactions with key residues in the active site. GF displays a pattern of interactions dominated by hydrogen bonds and hydrophobic interactions with crucial GPX-4 residues. The combination of these interactions, particularly the hydrogen bonds with lysine and methionine residues and the hydrophobic contacts with tryptophan suggests GF could stabilize within the active site, leading to inhibition of GPX-4 activity.

SVA also forms multiple hydrogen bonds and has significant ionic interaction with LYS48 (A), highlighting a strong affinity for the active site of GPX-4. The high interaction energy for the ionic bond (−5.8 kcal/mol) and an outstandingly low interaction energy for the most potent hydrogen-acceptor interaction with LYS48 (A) (−15.6 kcal/mol) illustrate the promising inhibitory capacity of SVA.

Given the relatively low docking scores for the GF compound, their interactions with GPX-4 suggest a potential for inhibition, with GF showing a slightly lower docking score compared to SVA. The presence of strong interactions within reasonable distances from the active site residues supports the potential inhibitory effect on GPX-4.

#### 5α-Reductase

Table 5 presents a comprehensive analysis of the interactions between GF and SVA with the enzyme 5α-Reductase, using its crystal structure (PDB: 3G1R). Docking scores are provided as a measure of each compound’s affinity and potential inhibitory effect on the enzyme. GF displays a hydrogen-acceptor interaction with NH1 of ARG134 (B) and a pi-pi stacking interaction with the 5-ring of TRP230 (B), resulting in a strong docking score of −8.7185 kcal/mol. SVA exhibits multiple powerful hydrogen-acceptor interactions with ARG134 (B) and significant ionic interactions at the same site, coupled with hydrophobic H-pi interactions with adjacent 6-rings. The calculated docking score for SVA is −9.2775 kcal/mol, suggesting its potential as a more potent inhibitor than GF against 5αRD. Table 5 and Fig. 6E,F are the summary of molecular docking interactions of GF and SVA with the active site residues of 5α-RD (5αRD, PDB: 3G1R). The table includes the type of interaction, interatomic distances, interaction energies, and docking scores for each compound.

**Table 5:** Summary of molecular docking interactions of Gefitinib and Tenivastatin with the active site residues of 5α-Reductase (5αR, PDB: 3G1R)

Tested molecule	Ligand	Receptor	Interaction	Distance	E (kcal/mol)	Docking score (kcal/mol)
Gefitinib	N14	NH1 ARG134 (B)	H-acceptor	3.19	−0.4	−8.7185
	6-ring	5-ring TRP230 (B)	pi-pi	3.88	0	
Tenivastatin	O8	NH1 ARG134 (B)	H-acceptor	2.94	−6.9	−9.2775
	O8	NH2 ARG134 (B)	H-acceptor	2.93	−2.6	
	O8	NH1 ARG134 (B)	Ionic	2.94	−4.9	
	O8	NH2 ARG134 (B)	Ionic	2.93	−4.9	
	C11	6-ring TYR132 (B)	H-pi	4.48	−0.4	
	C24	6-ring TRP230 (B)	H-pi	3.65	−0.4	
	C32	6-ring TYR132 (B)	H-pi	3.87	−0.5	
	C39	6-ring TYR26 (B)	H-pi	3.33	−0.3	

Both GF and SVA show promising interactions with key residues within the active site of 5α-RD, an enzyme implicated in androgen-associated disorders. The docking study reveals potential inhibitory effects based on the types of interactions and the calculated docking scores. For GF, although there is a lack of diversity in the types of interactions, the presence of a pi-pi stacking interaction with tryptophan suggests that it might interact tightly with the active site. The hydrogen-acceptor interaction with ARG134 further supports the notion that GF could potentially inhibit enzyme activity by binding strongly to the active site and affecting substrate accessibility or enzyme conformation.

SVA demonstrates a greater variety of interactions and markedly higher affinities, as suggested by the stronger interaction energies with ARG134, indicative of a robust binding potential. The hydrogen-acceptor interactions, along with ionic interactions at the same site, strongly imply that SVA could competitively bind to the active site, potentially preventing the binding of natural substrates. Furthermore, the additional H-pi interactions with aromatic residues may contribute to stabilization within the active site, further enhancing inhibition. The higher docking score for SVA (−9.2775 kcal/mol) in comparison to GF (−8.7185 kcal/mol) reflects its stronger overall potential to inhibit 5α-Reductase. Notably, SVA interactions are not only numerous but also include a significant ionic bond which greatly contributes to its binding strength.

## Discussion

Drug repurposing has been documented as a strategy for redirection of medical applications of approved drugs. Drug repurposing strategy accelerated the development of innovative oncotherapy [39]. In this regard, several studies repurposed statins and other lipid-lowering agents for cancer therapy. Specifically, SV was demonstrated as monotherapy or a combination therapy for the treatment of PCA [40]. Moreover, nanocarriers are authorized as cargoes for blending tumor medicines to target PCA cells [39]. This could bring anticancer medicines to control cancer metastasis [41]. Specifically, LNs could mediate the selective deposition of anticancers in the lymphatic system to combat tumor cell metastasis. The size of fenestrations of lymphatic vessels can reach about 500 nanometers or more in cancer conditions [41,42]. Thus, in the tumor microenvironment, the enlarged fenestrations of lymphatic vessels can facilitate lymphatic metastasis and the spread of cancer [41,42]. These enlarged fenestrations enable the delivery of LNs into the lymphatic system to combat cancer metastasis [41,42]. Specifically, ERLN can be activated by enzymes that are overexpressed in cancer cells. Particularly, esterase-responsive nanoparticles are suggested to deliver anticancer agents into tumor cells [17]. This study was conducted to develop ELNGFSV to enhance the cytotoxicity of GF and SV on PCA cell line.

### Influence of ERLN composition

The lipid-based nanoparticles have a propensity to the lymphatic system as a track for their journey in the body. However, ERLN mimics lipoproteins [42]. Interestingly, the porousness of lymphatic vessels in cancer cells is greater than in normal cells [42]. Thus, the gaps between lymphatic endothelial cells can reach up to 500 nm or more in malignant conditions [42]. In this context, several studies reported that lipid nanocargoes are lymphotropic agents. Thus, the size of ERLN is a vigorous factor in the import of drugs into tumor cells by the lymphatic system gateway [42].

ZP has an impact on drug loading, drug release, stability, cellular transport, and subcellular accumulation of drug-loaded LNs. The value of ZP more than negative 30 mV and up to positive 30 mV is desired for the stability of ERLN [43,44]. Otherwise, LNs that have low ZP values smaller than 5 mV tend to agglomerate, while extreme ZP values above 30 mV lead to the monodispersity of ERLN [45]. Accordingly, the engineering of the LN surface is crucial to control stability, cellular uptake, and therapeutic impact. Certainly, the modified LNs can selectively transport the medicine into the cellular and subcellular compartments.

ERLN that have negative to neutral ZP could permeate the mucus gel layer, but, nanocarriers exhibiting a positive ZP are entrapped in the mucus layer by electrostatic interactions with the negative charge of mucus due to sialic acids. Thus, the transport of nanocarriers by epithelial cells is favored by a positive charge [45]. The ZP of LNs could be engineered by the use of surfactants, and polymers to provide the desired electrostatic characteristic of LNs. Furthermore, ZP is affected by the nature of the solution, such as pH and ionic strength [45]. Likewise, several studies demonstrated that CS decoration shifts the ZP of LNs into positive ZP with enhanced cellular uptake, bioavailability, and effectiveness [28,29].

LNs engineered phospholipids to act as substrates for phospholipase [24]. Thus, ERLN could promote the delivery of anticancer agents into tumor cells selectively [17]. In the present study, lipoid S100 was added to the phosphorylcholine layer on the exterior surface of ERLN.

In the tumor microenvironments, the prepared ERLN cargoes can release their drug payload under the effect of esterases such as lipase, and phospholipase enzymes overexpressed by cancer cells [46]. In the present study, the surface of ERLN was engineered by triacylglycerol, and phosphatidylcholine as substrates for hydrolytic enzymes. Likewise, several studies reported that esterase-responsive nanoparticles promote the selective liberation of drugs at the tumor site [17,25].

### Biocompatibility of ELNs

Commonly, the hemolytic effect of ERLN on RBCs is used as an indicator of biocompatibility [47]. Thus, biocompatibility refers to the biosafety of ERLN to deliver the drug to the desired destination without damaging effects on biological systems [47]. Moreover, biocompatibility studies are essential to exclude the effect of additives such as polymers, surfactants, lipids, or other ingredients of ERLN. Several studies indicated that ERLN that induced 5% lysis of RBCs are acceptable for biological studies [48]. In the current study, the hemolysis percent of DMSO, GF, SV, and GFSV combination, as well as PELN, ELNGFSV, CPELN, and CELNGFSV was less than 10 percent, after 24 h. These findings are aligned with several studies that established the biocompatibility and safety of ERLN on RBCs [47,48]. As well, another study indicated the biosafety of ERLNs with hemolytic effect on RBCs less than 5% percentage [49]. The gentle effect of ERLN on RBCs indicates the precise amount of ingredients of ERLN. Consequently, the present results are synchronized with several studies that investigated the hemocompatibility and biosafety of LNs [21]. Thus, PERLN, ERLNGFSV, CPERLN, and CERLNGFSV are appropriate and could be intended for cytotoxicity studies on PCA cell line.

### ERLNGFSV-induced PC-3 cell mortality

The present findings confirmed the synergistic effect of GF plus SV codelivery on PC-3 death compared to control, free GF, and SV. Additionally, ERLNGFSV and CERLNGFSV induced an increase in PC-3 cell death increases by increasing the incubation time. Likewise, several studies demonstrated that the GF and SV combination as well as GFSV-loaded LNs induced a synergistic effect on cancer cell death. In this regard, ample studies indicated that the combination of statins and TKIs boosted cancer cell death [50]. Moreover, statins are documented to induce synergistic effects with other anticancer agents [51]. Thus, the combination of GF plus SV induced a synergistic effect on PC-3 cells in the present study. This effect is attributed to their inhibitory action on kinases affecting growth signal cascades.

Remarkably, the cytotoxic effect of ERLNGFSV on PC-3 cells was augmented by CS capping CERLNGFSV. Likewise, another study indicated that drug-loaded LNs expand the therapeutic influence of cytotoxic drugs [52]. Moreover, SV-loaded CS nanoparticles enhance cancer cell killing [15,31]. The cationic ZP of ERLN due to the presence of CS could enhance the drug uptake by the tumor cell [15]. The interaction between the cationic ammonium group of CS and the negatively charged groups on the cell surface of cancer cells augments the cellular uptake and cytotoxicity of GF plus SV [53]. Also, CS could elicit a cytotoxic effect by reducing cholesterol availability for the building of cancer cells [28]. Moreover, CS elicits cytotoxicity by plasma membrane damage and organelles damage. Due to the presence of cationic ZP, CERLNGFSV still has the propensity to target cell nuclei and interact with DNA and RNA, therefore replication and transcription of DNA were blocked with a reduction of cancer cell viability [54].

Collectively, the present results confirmed the finding indicated by several studies that documented statins as monotherapy, combined therapy with GF or other anticancer agents inhibit the growth of PCA [55]. Similarly, several studies documented that concurrent therapy of SV with chemotherapies induced a synergistic effect on cancer cell death. Moreover, SV therapy sensitized the cancer cells to anticancer drugs with an improved therapeutic impact [56]. Additionally, SV is suggested to decrease of MDR of PCA cells to chemotherapy. This effect results from the down-regulation of cholesterol and small G-proteins [57]. In this context, it has been reported that SV reduces the growth of PCA xenograft tumors [57]. As well, several studies reported that statins alone exert antitumor effects and augment the response of PCA to chemotherapy by encouraging apoptosis and cell cycle arrest of PCA cells [13].

### ERLNGFSV-induced PC-3 apoptosis

The induction of apoptosis, autophagy, ferroptosis, and pyroptosis is suggested as a mechanism to provoke the cytotoxic effect of anticancer therapy [13]. In the current study, the percentage of PC-3 apoptosis was increased after treatment with GF, SV, GF+SV, ERLNGFSV, and CERLNGFSV compared to control PC-3 cells. The present results agree with several studies reported that GF plus SV could induce apoptosis, ferroptosis, and pyroptosis as mechanisms for death [13]. Moreover, treatment of PC-3 cells with GF plus SV, ERLNGFSV, or CERLNGFSV elicits a marked increase of apoptotic cells compared to other groups. These findings agree with several studies that document the effect of GF and SV on the cancer cell programmed cell death. In this context, a previous study demonstrated that GF-induced apoptosis and cell cycle arrest of PCA and other cancers by disruption of multiple signaling pathways that mediate the malignant transformation of PCA [58]. Likewise, GF treatment increases mitochondrial permeabilization, cytochrome C release, and activation of several caspases which are crucial for programmed cell death [58]. Accordingly, GF-induced apoptosis significantly reduces the viability of PC-3 cells. Also, SV inhibits the mevalonate pathway that is essential for the prenylation of small GTPase that plays a vital role in cell survival, proliferation, migration, and apoptosis [59].

Additionally, SV induces apoptosis through both intrinsic and extrinsic pathways [59]. Thus, a combination of GF plus SV targets different pathways involved in cell survival, proliferation, and apoptosis potentially leading to a synergistic effect on PCA death [59]. In this context, the present study confirmed that the GF and SV combination significantly increased apoptosis in PC-3 cells compared to free drugs due synergistic effect of several signaling pathways involved in apoptosis. In this regard, in a previous study, GF and SV codelivery enhanced apoptosis in GF-resistant lung cancer cells [59].

In the present study, CERLNGFSV elicited a marked effect on PC-3 cell apoptosis compared to other groups. This effect may be attributed to the presence of CS. These findings are concurrent with several studies indicating the effect of CS on the apoptosis of cancer cells [15]. Thus, the CS cationic coat on ERLNs provokes electrostatic interaction with cancer cell membranes [43]. This induced an obvious increase in cancer cell expiry [29]. Moreover, CS chelates cholesterol, which decreases the membrane assembly of tumor cells [29]. Likewise, CS-cationic corona on ERLN augments cellular uptake of anticancer drugs into cancer cells [29]. Similarly, our previous study established that CS-decorated SV-loaded nanoparticles provoked cancer cell apoptosis [15].

Collectively, loading GF and SV ERLN can enhance their delivery to cancer cells, increasing their intracellular concentration and efficacy [60]. However, nanoparticles can facilitate the co-delivery of the drugs, ensuring that they reach the target cells simultaneously and at optimal ratios [31]. In this context, developed ERLN loaded with GF plus and SV enhanced delivery and efficacy in inducing apoptosis in PC-3 cells. In this regard, the nanoparticles improved drug stability and bioavailability, and enhanced induction of apoptosis [61]. MDS results revealed that GF and SV might disrupt the mitochondrial function due to SDH inhibition. The mitochondrial malfunction is associated with the release of pro-apoptotic proteins that could mediate PCA cell death.

Moreover, mitochondrial malfunction decreases ATP production. As well, mitochondrial dysfunction induced redox imbalance and cancer cell death [62]. Moreover, inhibition of GPX-4 promotes the ferroptosis in tumor cells [13]. Likewise, the inhibition of 5α-RD reduces the production of androgen as a risk for PCA [63].

### MDS of GF and SV with SDH, GPX-4, and 5α-RD

In the present work, MDS of GF and SV with SDH, GPX-4, and 5α-RD provides new insight into the use of these drugs for the treatment of PCA. These enzymes suggest targets to explain the cytotoxic effect of GF and SV on PCA cell death. Moreover, GPX-4 is an antioxidant enzyme, it is one of the factors influencing the regulation of ferroptosis. The induction of ferroptosis in tumor cells is a promising anti-tumor strategy [13]. Likewise, the inhibition of 5α-RD is essential for the conversion of cholesterol androgen and has the potential for the discovery of anticancer agents [64]. Collectively, the binding of GF and SV with SDH, GPX-4, and 5α-RD explains the synergistic cytotoxic effect of GF and SV on the PC-3 cell line.

Indeed, the lipids content of LNs promotes their uptake by highly proliferative cancer cells [64]. Therefore, ERLNs mediate the concurrent delivery of GF and SV into the tumor cells by passive and active machinery [20]. Thus, ERLNs could imported into cancer cells by lipid receptors overexpressed on cancer cell surfaces [21]. For example, PCA cells overexpress LDLR to compensate for the high cholesterol as a precursor of steroid hormones, besides, the increased endogenous biosynthesis of cholesterol [56]. The abnormal cholesterol homeostasis is a contributing factor to MDR by cancer cells [57], however, high cholesterol modifies the membrane functions, besides modulation the function of several proteins involved in cancer cell growth signal [57]. Consequently, the diminution of cholesterol by SV could inhibit the activity of kinases, and multiple oncogenic signals involved in malignant transformation of PCA [55]. In this regard, the antiproliferative effect of SV could explained by influencing the function of small GTPases, CoQ10 production, cytochromes, and androgens production, hence, SV inhibits PCA cell proliferation [57]. Specifically, statins can reduce steroid hormone production and thus represent a promising approach for the cancer therapy of PCA [55,63]. Likewise, several studies confirmed the targeting of cholesterol biosynthesis, uptake, efflux, and storage cloud suppresses PCA cell growth [56].

Therefore, SV is suggested as chemo-preventive, monotherapy for cancer, or in combination with GF to enhance cancer cell death [65]. Consequently, the treatment of PCA cells with GF plus SV induced a synergistic cell death and inhibited MDR of PCA cells [66]. The synergistic effect of GF and SV may be attributed to the similarity in the mechanism of action on kinases. Additionally, GF and SV are the substrates of the same metabolizing enzymes.

Thus, the treatment of PC-3 cells with GF plus SV inhibits multiple therapeutic targets dealing with MDR PCA cells. In this context, codelivery of GF and SV in ERLNs is a promising approach for overcoming the MDR. Likewise, ample studies recommended esterase-responsive drug cargoes for selective cancer cell drug release [67]. Moreover, esterase-responsive nanoparticles inhibit tumor growth with low side effects [67].

This study has numerous restrictions. Initially, this study mainly relies on *in vitro* experiments using PC-3 cells. Another is the study’s attention on short-term cellular effects, without examining potential long-term significances in the context of MDR studies. Third, while molecular docking provides forecasts on drug interactions with SDH, GPX-4, and 5α-RD, these interactions have not yet been validated through biological studies. Future research will include further studies using several PCA cell lines to validate the present findings. Furthermore, *in vivo* studies are crucial to fully assess the potential efficacy of the developed ERLNs in treating PCA.

## Conclusion

The present study concluded that the ultrasonic melt-emulsification technique produced colloidal, nanoscale, homogenous, and biocompatible ERLNs. Based on the composition, PS, ZP, and PDI of ERLNs, they are imaginary lymphatic drug delivery systems. Coconut oil, lipoid S100, and CS were used to form a triple shell on ERLNs. CS decoration induces cationic corona on ERLNs to mediate the cellular uptake of ERLNs. The components of ERLN in terms of triacylglycerol and phosphatidylcholine could act as substrates for lipases and phospholipases that are overexpressed in PCA cells. Therefore, the drugs are selectively released in PCA cells compared to normal cells Moreover, the nanosized, cationic ZP and composition of ERLN could facilitate the selective codelivery and lymphatic accumulation of GF and SV into PCA cells. MDS studies revealed varying affinities of GF and SVA for binding with different molecular targets SDH, GPX-4, and 5α-RD. Thus, GF plus SV, ELNGFSV, and CELNGFSV treatment induced synergistic PC-3 cell death compared to control PC-3 cells due to targeting multiple cascades involved in PC-3 cell growth. Future studies using several PCA cell lines are required to validate the present findings. As well, *in vivo* studies are essential to establish the influence of CELNGFSV on PCA.

## Data Availability

All data generated or analyzed during this study are included in this published article.
